# Deuterium 1‐Channel Transmit/16‐Channel High Impedance Receive Array Combined With 16‐Channel ^1^H Dual‐Row Transceiver Array for 7 Tesla Brain Imaging

**DOI:** 10.1002/mrm.70268

**Published:** 2026-01-23

**Authors:** Bei Zhang, Wenkai Liang, Chichen Dong, Anke Henning

**Affiliations:** ^1^ Advanced Imaging Research Center University of Texas Southwestern Medical Center Dallas Texas USA

## Abstract

**Purpose:**

To develop and evaluate an ^2^H/^1^H coil configuration that enables deuterium metabolic imaging at 7 T while preserving high‐quality ^1^H anatomical imaging.

**Methods:**

An 16‐channel ^2^H high‐impedance receive array was combined with a ^2^H transmit birdcage and a 16‐channel dual‐row ^1^H transceiver array. Electromagnetic simulations are used to assessed B_1_^+^ efficiency and SAR. Phantom and in vivo experiments were performed on a Philips 7T system to evaluate transmit efficiency, signal‐to‐noise ratio, and signal coverage.

**Results:**

Simulations results match with experimental ones, and they demonstrated good transmit efficiency, receive sensivity and image quality. In vivo 2H 3DMRSI map demonstrated whole brain coverage.

**Conclusion:**

The proposed coil configuration enables robust 2H metabolic imaging at 7T while preserving good 1H performance.

## 1 Introduction

Deuterium metabolic imaging (DMI) has recently emerged as a prominent area of research in MRI community [1, 2, 3]. This innovative and noninvasive technique enables the direct observation of metabolic fluxes in tissues, such as glucose metabolism and fat oxidation, through the administration of deuterium‐labeled substrates [3, 4, 5, 6, 7, 8, 9]. Furthermore, DMI facilitates real‐time studies of metabolic pathways, including energy metabolism and pharmacokinetics, providing valuable information on biochemical dynamics in vivo [5, 10].

Due to the naturally low abundance [11] and low gyromagnetic ratio of deuterium (*γ* = 6.536 MHz/T), its signal is substantially weaker compared to that of protons (*γ* = 42.577 MHz/T). As a result, many DMI studies are conducted at ultrahigh fields (UHF), which offer a high intrinsic signal‐to‐noise ratio (SNR) [1, 11]. However, designing dual‐tuned ^2^H/^1^H RF coils, an essential tool for DMI, presents significant challenges. At UHF, the short wavelength at the ^1^H frequency leads to strong electromagnetic interference between the ^2^H and ^1^H coil elements, similar to other multinuclear coil designs. An early common solution was adding ^1^H LC traps on the multinuclear coil elements to reduce interference [12, 13]. These traps introduced extra conductive losses. Meyerspeer et al. improved the ^1^H trap design by replacing the LC trap with an LCC one, which reduced conductive losses from 30% to 10% in their single surface coil design [14]. In large surface coils or volume coils, however, more than one LCC trap are required per coil element [15], further diminishing the SNR of multinuclear coils. This posed a particular challenge for ^2^H/^1^H coil designs, where maintaining good ^2^H SNR is critical for DMI studies.

Initially, volume coils were predominantly used in ^2^H/^1^H coil development due to their ability to provide uniform signal coverage and simplicity in design [16]. However, these coils were not optimized for maximizing SNR. Recent advancements have shifted toward phased arrays for ^2^H acquisition, especially at ultra‐high magnetic fields, to enhance SNR. For example, Soon et al. introduced an 8‐channel ^2^H/^1^H transceiver array utilizing LC tanks to enable resonance at both ^1^H and ^2^H frequencies [17]. While effective for dual‐resonance functionality, these LC tanks introduced additional conductive losses, reducing the SNR for ^2^H. To address this issue, Ruhm et al. developed a 9.4T ^2^H/^1^H array [9], which comprised eight ^1^H coil elements and eight separate ^2^H coil elements. An inductive decoupling mechanism was employed to isolate the ^2^H coil elements, and multiple LCC traps were incorporated to mitigate cross‐interference. However, both the decoupling mechanism and the LCC traps contributed to additional conductive losses, compromising the ^2^H SNR. In both designs, the ^2^H coil elements functioned as transceivers, serving dual roles in signal transmission and reception.

In 2012, Qian et al. demonstrated that a 7T 15‐channel receive array provides superior SNR than a birdcage for brain sodium imaging [18], even though it can be seen from the noise correlation matrix that the coil elements are strongly coupled with each other. SNR with noise decorrelation showed a significant improvement over the SNR without. However, this design was limited to sodium imaging only and did not have ^1^H MRI capability, hence lacking the capability to perform calibration steps such as frequency determination or B_0_ shimming and did not allow for proton and sodium imaging without change of RF coil. More recently, a commercial 7T 32‐channel sodium receive‐only array (RAPID Biomedical) demonstrated significant SNR improvement over a birdcage coil [19], highlighting the advantages of separating transmit and receive coil elements in multinuclear designs. This dual‐tuned ^23^Na/^1^H coil was capable of both ^23^Na MRSI and ^1^H MRI, though its design details have not been disclosed.

Despite these advancements, no 7T deuterium receive arrays have been reported to date. In this work, we address the gap by developing a 7T 16‐channel ^2^H receive array featuring high‐impedance coil elements which show excellent decoupling performance in phased array [20]. This array is paired with a detunable ^2^H transmit birdcage coil and a 16‐channel ^1^H transceiver array, specifically for DMI of the brain at 7T. This configuration aims to achieve good ^2^H and ^1^H image quality [21, 22].

## 2 Methods

**FIGURE 1:**
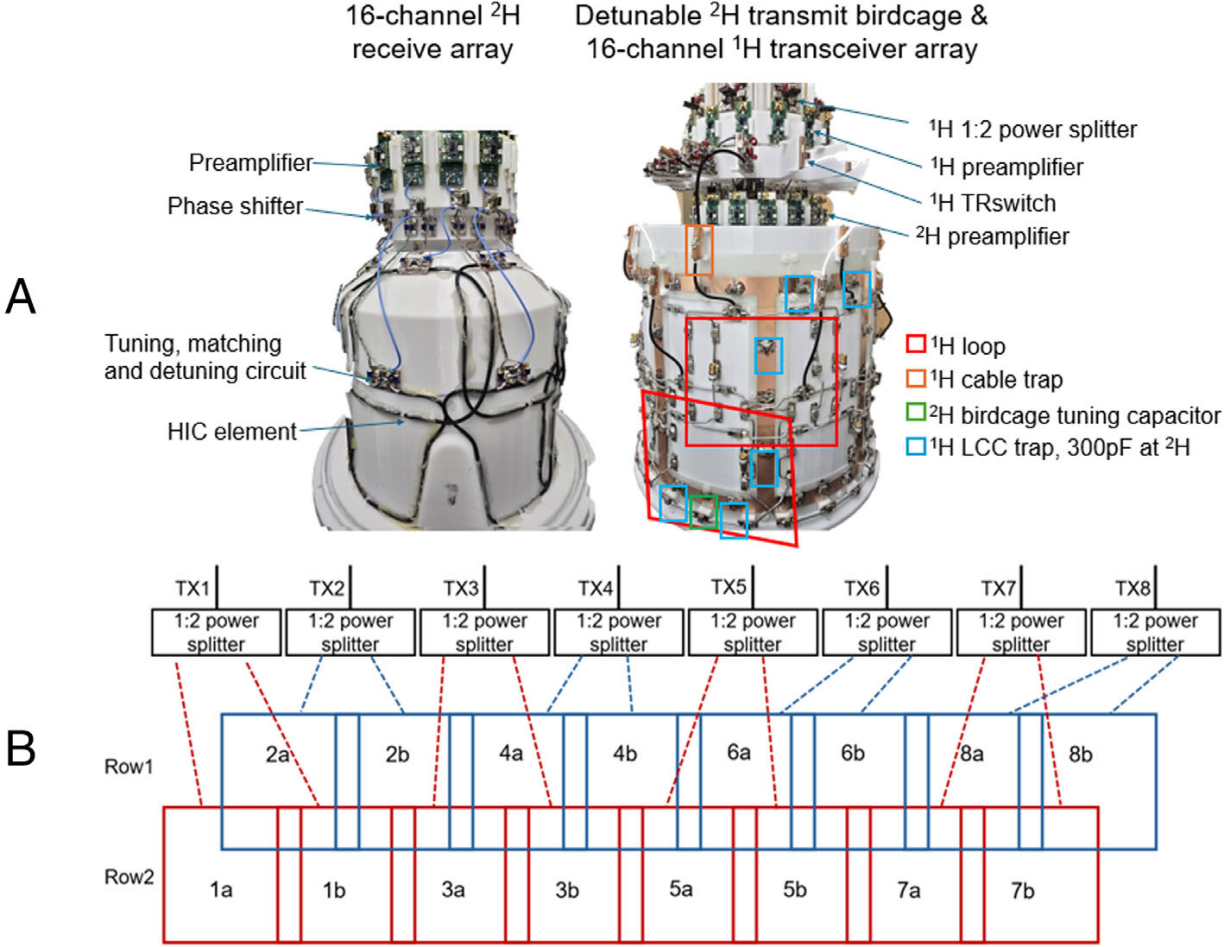
(a) Pictures of the 7T 16‐channel ^2^H receive array sitting on the inner layer (left), it includes the 16 ^2^H HIC elements, tuning, matching, and detuning circuit boards, ^2^H preamplifiers, as well as phase shifters and coaxial cable for preamplifier decoupling; and the detunable ^2^H bandpass transmit birdcage and dual‐row ^1^H 16‐channel transceiver array (right), wherein different modules and components were indicated by squares of different colors. (b) 2D array configuration for ^1^H 16‐channel transceiver array.

Figure 1a shows images of the coil prototype. The coil housing was designed using computer aided design (CAD) software SolidWorks and fabricated via a 3D printer (Fortus MC450, Stratasys) with polycarbonate material. The housing components have a uniform thickness of 3 mm. The structure comprises two concentric layers. The outer layer is a cylindrical shell with a diameter of 270 mm and a length of 280 mm. The inner layer consists of a close‐fitting helmet shaped as an elliptic cylinder measured 220 mm (left–right) × 234 mm (anterior–posterior) × 180 mm (head‐feet), combined with a 40 mm‐long conical frustum on the dome area. An additional 15 mm extrusion was added to accommodate the nose. The ^2^H 16‐channel receive array was positioned on the inner layer, while the ^2^H transmit birdcage and ^1^H 16‐channel transceiver array were mounted on the outer layer.

### 2.1 ^2^H Receive Array Design

**FIGURE 2:**
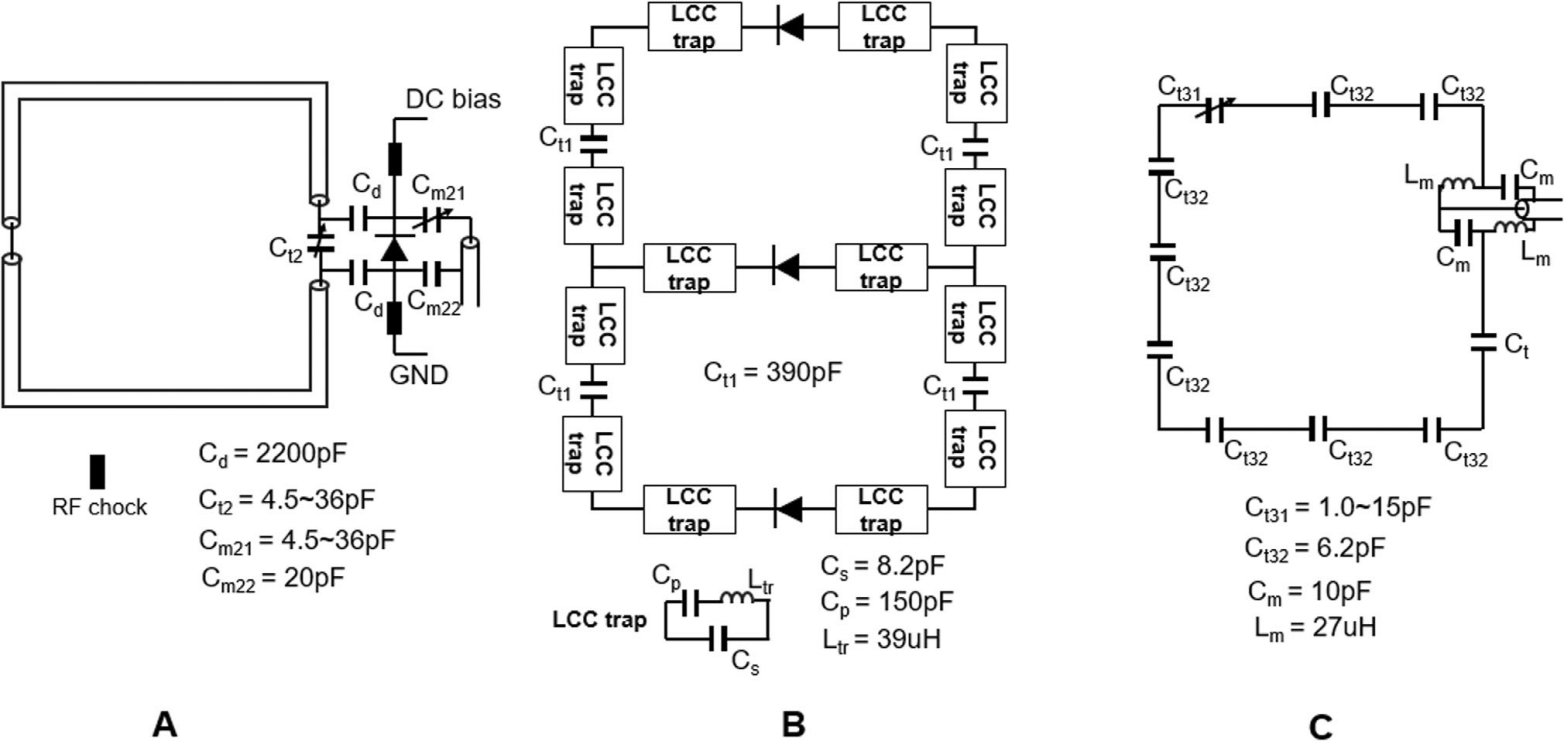
(A) Schematic diagram of the ^2^H HIC element, *C*_*t2*_ is used for tuning, *C*_m21_ and *C*_m22_ for matching, and PIN diode for the active detuning circuit. (B) Schematic diagram of the ^2^H transmit bandpass transmit birdcage, two segments of both end‐rings and three rungs were illustrated, there are two ^1^H LCC traps on each end‐ring segment and two on each rung, one high‐power PIN diode was placed on each rung for detuning the ^2^H transmit birdcage during ^2^H receive and ^1^H transmit and receive. (C) Schematic diagram of the ^1^H transceiver loop, 10 tuning capacitors were distributed on the loop and a lattice balun is used for matching.

The ^2^H receive array consisted of 16 high impedance coil (HIC) elements [23, 24]. Each HIC element was constructed using a 450 mm‐long 25.4 mm‐diameter 50 Ω coaxial cable (Molex 100 066‐0054). All the coil elements were affixed to the inner layer in a dual‐row 2 × 8 layout. Random overlap was applied between adjacent coil elements to achieve a smooth receive sensitivity profile, except for the two coil elements near the nose, which had no overlap due to the limited space between the noise extrusion area and the outer layer. The schematic diagram of the HIC element is shown in Figure 2A. Tuning, matching, and active detuning circuits were integrated onto a compact 18 mm × 26 mm printed circuit board (PCB). Tuning to 45.6 MHz was achieved using the capacitor *C*_t2_ (Knowles Voltronics, JZ300HV), while matching to 50 Ω was achieved by capacitors *C*_m21_ (Knowles Syfer, serial 1111) and *C*_m22_ (Knowles Voltronics, JZ300HV). Active detuning was implemented by a PIN diode and RF chokes. After matching, each HIC element was connected to a preamplifier (WanTcom, WMM46P) through a phase shifter (75° for the top row to 78° for the bottom row) and interconnecting coaxial cable, whose length was adjusted for preamplifier decoupling.

### 2.2 ^2^H Transmit Coil

The ^2^H transmit coil was designed as an eight‐rung bandpass transmit birdcage with a length of 220 mm along the *z*‐axis. Both the rungs and end‐rings were fabricated using 15‐mm‐wide copper strips. Figure 2B illustrates the schematic diagram of the bandpass transmit birdcage. To minimize its interference with the ^1^H array, two ^1^H LCC traps (˜300 pF at ^2^H) were positioned on each rung, along with two additional traps on each end‐ring segment [13]. Because these 1H LCC traps are equivalent to 300 pF capacitors at ^2^H frequency, it is considered that there are capacitors on both end rings and rungs. Thus, the 2H transmit birdcage functions as a band‐pass birdcage, with an effective capacitance of ˜150 pF on each rung (300 ∥300 pF) and ˜108 pF on each end ring segment (300∥390∥300 pF). Detuning was achieved by placing a high‐power PIN diode at the center of each rung, so that the transmit birdcage only resonates at 45.6 MHz during ^2^H transmit, when a 100 mA DC bias current was applied to the PIN diodes. The 0° and 90° ports were located on the end‐ring near the dome, positioned symmetrically in the middle of two adjacent segments next to the top one. A ^2^H quadrature hybrid was used to drive the ports, creating a circularly polarized (CP) B_1_^+^ field.

### 2.3 ^1^H Transceiver Array

The ^1^H array was positioned with a 20 mm offset toward the feet relative to the ^2^H transmit birdcage along the *z*‐axis. The array featured a dual‐row 2 × 8 loop configuration, with each loop measured approximately 142 mm × 115 mm. Each loop was constructed using 12‐AWG (2.05 mm in diameter) copper wire. The two rows were offset by 45° in *x*‐*y* plane, and geometric overlap was implemented by bending the copper wires to achieve decoupling between neighboring coil elements on workbench. Its 2D Array configuration is shown in Figure 1b. The loop was segmented into 11 sections, with one variable capacitor and nine fixed capacitors (6.2 pF each, Knowles Syfer, 2225 series) placed at the segments to tune the loops to 298 MHz. A lattice balun was used for matching in the middle of the last segment. All 16 loops functioned as transceivers. After matching, each loop was connected to a ^1^H transmit/receive (TR) switch [25], with the cable length between the TR switch and the preamplifier (WMM7RP) adjusted for preamplifier decoupling of each coil element. A ^1^H shielded inline cable trap was placed at every ^1^H ¼ λ along the coaxial cable connecting the coil to the TR switch. The ^1^H array was driven by eight individual transmit channels. In each row, two neighboring loops shared a single transmit channel through a 1:2 power splitter with a 45° geometric phase offset. Figure 2C illustrates the schematic design of a single ^1^H loop. During ^1^H TR, both the ^2^H transmit birdcage and receive array were detuned and remained off.

**FIGURE 3:**
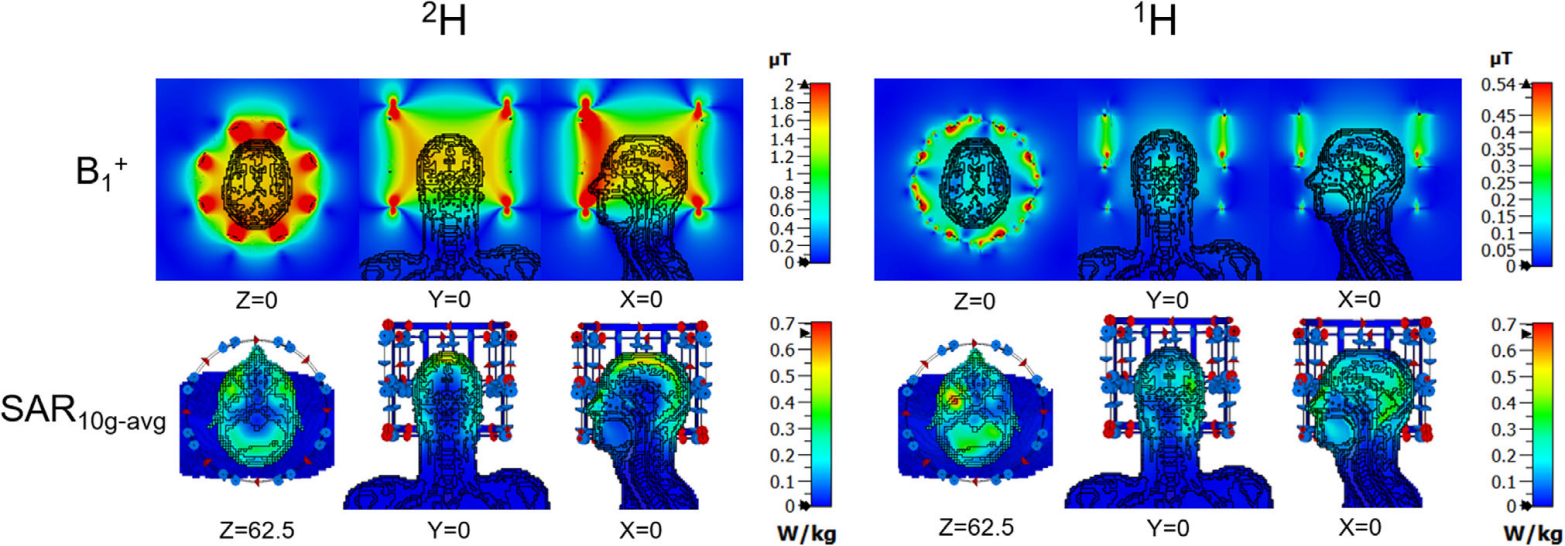
Simulated B_1_^+^ (per 1 W input power) and SAR distribution (per 1 W accepted power) of the ^2^H transmit birdcage and the ^1^H 16‐channel transceiver array on the truncated Duke model.

To assess the safety parameters of the array for practical applications, full‐wave simulations were conducted using CST Microwave Studio (Computer Simulation Technology, Darmstadt, Germany). The ^2^H transmit birdcage and ^1^H transceiver array were modeled with dimensions identical to their physical prototypes and loaded with a 5 mm isotropic Duke brain model [26]. The dielectric properties of the brain tissue were set to match those of the human brain at 298 MHz, as shown in Figure 3. CST co‐simulation tool was employed for tuning and matching the respective ^1^H and ^2^H coils. B_1_^+^ per watt of accepted power and the 10 g‐averaged specific absorption rate (SAR) were computed using CST's post‐processing module. These results were used to define SAR‐related parameters on the scanner, following the vendor's SAR configuration guidelines.

Phantom experiment was conducted to verify the spatial coverage and transmit efficiency of the ^1^H 16‐channel transceiver array, ^1^H B_1_^+^ maps were acquired with an AFI sequence [27] on a head and neck phantom (17″ *H* ×12″ *W* × 9.5″ *D* with a circumference at the brow of 22″ approximately), whose relative permittivity is 62 and conductivity is 0.45 S/m. The experimental B_1_^+^ maps were compared with the simulated ^1^H B_1_^+^ on duke. The AFI sequence parameters are resolution 2.0 mm × 2.0 mm, slice thickness 5.0 mm, FOV 240 mm × 240 mm, FA of 60°, Act. TR/TE 100/1.98 ms, min. TR/TE 9.7/1.98 ms, and the number of averages 2.

Since the head and neck phantom does not contain deuterated water, we built a 2 L 12 cm‐diameter cylindrical 4% deuterated water phantom to evaluate the ^2^H B_1_^+^ and SNR in experiment. The dielectric properties of the phantom are *ɛ*_*r*_ = 57, *σ* = 0.53 S/m. To ensure the ^2^H transmission performance, the same ^2^H transmit birdcage and ^1^H transceiver array loaded with the ^2^H phantom were modeled to compare the simulated ^2^H B_1_^+^ with the experimental one. Neither tuning nor matching of the ^2^H transmit birdcage and the ^1^H transceiver array was readjusted in simulation, similar to the experimental setup. B_1_^+^ efficiency per watt of simulated power was computed using CST's post‐processing module. The simulated ^2^H B_1_^+^ were compared with the experimental ^2^H B_1_^+^ map, which was calculated with the double‐angle method [28], by acquiring a 3D MRSI with flip angles 30° and 60°, respectively. SNR calculations were performed voxel‐by‐voxel by dividing the absolute peak signal by the noise standard deviation (SD) which was calculated from the first 50 noise‐dominated spectra sample points of the same voxel from the MRSI data with 30° flip angle.

^1^H SNR Was Acquired From Two GRE Sequences by Switching On/Off the RF Power on the Head and Neck Phantom. The SNR was compared with the 8‐channel Tx array in the Nova 8Tx32Rx array, as the two coils have the same diameter. The 8‐channel Tx array can be used as receive as well in Philips 7T MRI scanner. The acquisition parameters are resolution is 1.0 mm × 1.0 mm, Slice thickness 5 mm, FA 8°, TR 300 ms, TE 3.73 ms, and FOV 240 mm × 240 mm. All in vivo imaging was conducted on a 7T whole‐body MRI scanner (Philips Healthcare, 7TdSync) equipped with eight individual 2 kW transmit channels, under an approved institutional review board protocol. A high‐resolution 3D MP2RAGE sequence was used to acquire T_1_ weighted images with a resolution of 1.0 mm × 1.0 mm × 1.0 mm, FOV of 256 mm × 256 mm × 256 mm, FA of 8°, TR of 1800 ms, and TE of 2.26 ms. Spectra of natural abundance of deuterated water in the brain were obtained using the above 3D MRSI sequence with parameters: FOV = 220 mm × 206 mm × 182 mm, voxel size = 14 mm × 14 mm × 14 mm, TR = 155 ms, 11 Hanning‐weighted cartesian sampled k‐space averages, rectangular pulse duration (*T*_p_) = 0.5 ms, flip angle = 51°, vector size = 512, acquisition bandwidth of 5000 Hz, and acquisition time = 9min 24 s. The ^2^H processing pipeline described in [9] is used to process the acquired raw data.

## 3 Results

The unloaded quality factor (*Q*) of a single ^1^H loop mounted on the outer layer, measured without the matching network and with the ^2^H transmit birdcage present, was 132.6. When the ^1^H loop and ^2^H transmit birdcage were loaded with the head‐and‐neck phantom, the loaded *Q* was measured as 58.4. When the ^2^H receive array was additionally placed inside the ^2^H transmit birdcage, the loaded *Q* of the ^1^H coil element increased slightly to 62.1. The minimal difference in loaded *Q* values indicates that the presence of the ^2^H array had a negligible impact on the performance of the ^1^H coil. For the 2H birdcage, the measured *Q*_unload_/*Q*_load_ ratios were 146/58 = 2.52 without the ^1^H LCC traps and 89/47 = 1.89 with. The isolation of the ^1^H TR switch between the transmit and receive ports was measured at −54 dB. For the ^2^H HIC elements, the tuning and detuning difference is more than 50 dB on workbench test. Furthermore, the preamplifier decoupling for the ^2^H HIC element was approximately −23 dB.

Figure 3 presents the simulated B_1_^+^ at the isocenter and SAR distributions at the *z* = 62.5 mm plane where the maximal 10 g‐averaged SAR is located for both the ^2^H transmit birdcage and ^1^H transceiver arrays. With 1 W input power, the ^2^H transmit birdcage coil achieved higher B_1_^+^ than the ^1^H transceiver array at the center of the brain and also demonstrated uniform excitation in the entire region of interest (ROI). Despite both transmit coils operating in CP mode, notable differences in transmit field homogeneity were observed: the ^2^H B_1_^+^ distribution was highly uniform across the brain, whereas the ^1^H B_1_^+^ field exhibited a peak at the central region and decreased toward the periphery as expected from the difference in resonance frequency of 45.6 MHz for ^2^H versus 298 MHz for ^1^H. SAR analysis showed distinct patterns for the two nuclei. The ^2^H transmit birdcage exhibited maximum SAR values in the peripheral regions near the dome of the head model. Conversely, the ^1^H transceiver array displayed maximal SAR values deeper within the brain tissue, aligned with its more focused B_1_^+^ distribution. Table 1 summarizes the maximal SAR values for the entire RF coil model, as well as the mean and standard deviation SD of B_1_^+^ over a 20 mm × 20 mm × 20 mm volume at the center. The ^2^H transmit birdcage exhibited a highly homogeneous distribution, with a SD of 0.026, whereas the ^1^H transceiver array had a significantly higher deviation of 0.502, indicating greater inhomogeneity.

**TABLE 1:** Simulated maximal 10 g‐averaged SAR of the whole Duke model, as well as mean and deviation values of B_1_^+^ over a central (20 mm)^3^ volume, per 1 W input power.

	Maximal 10 g‐averaged SAR (W/kg)	Mean B_1_^+^ (μT) over a central (20 mm)^3^ volume	Deviation of B_1_^+^ over the central (20 mm)^3^ volume
^1^H 16‐channel transceiver array	0.661	0.17	0.502
^2^H transmit birdcage	0.579	1.563	0.026

**FIGURE 4:**
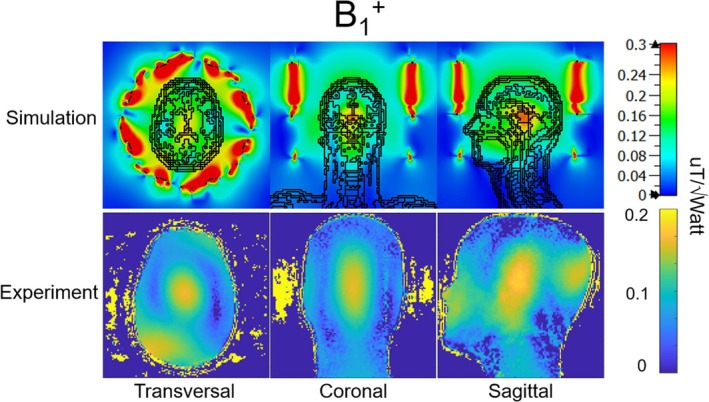
Simulated ^1^H B_1_^+^ of the ^1^H 16‐channel transceiver array on Duke and the experimental B_1_^+^ on a head and shoulder phantom, acquired with an AFI sequence.

Experimental ^1^H B_1_^+^ measurements on a head and neck phantom closely aligned with the simulated results, as shown in Figure 4. Both simulated and experimental results confirmed that the highest B_1_^+^ of the ^1^H transceiver array was located near the thalamus when the array was driven in CP mode using the geometric relationship of the 16 transceiver coil elements. With 1 watt input power, the B_1_^+^ field reached 0.17 μT in simulations and 0.174 μT in phantom experiment at the center of the brain.

**FIGURE 5:**
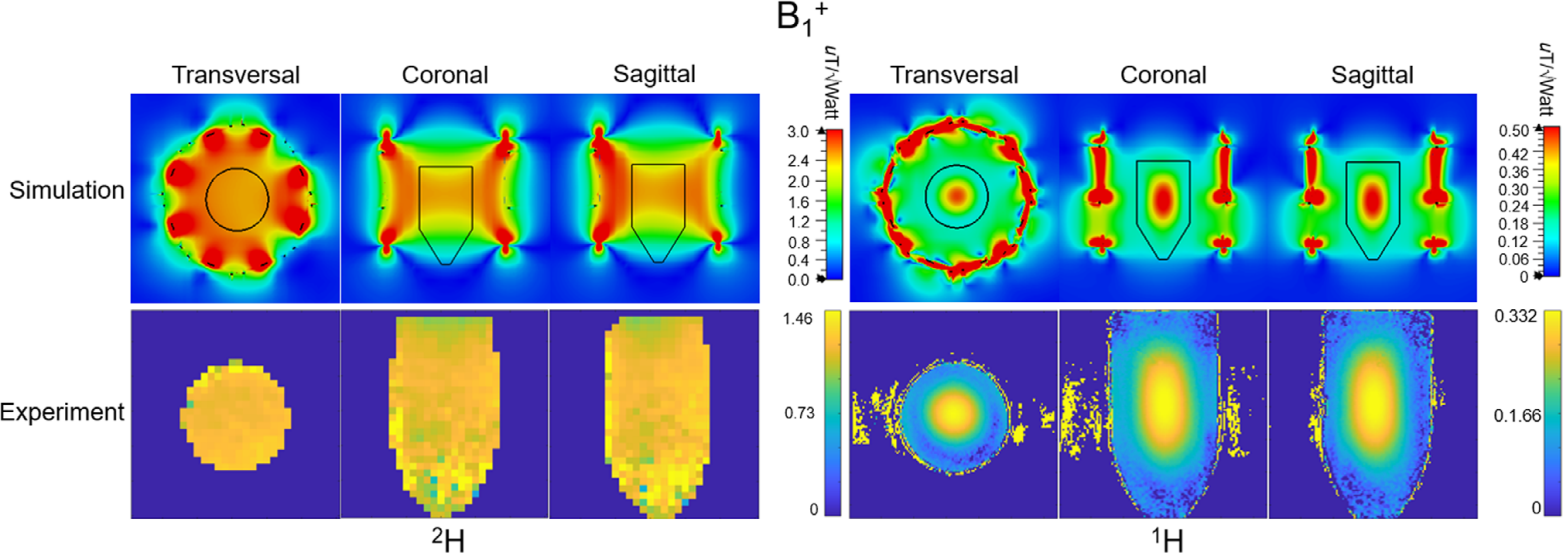
Simulated and experimental ^2^H and ^1^H B_1_^+^ of the 7T ^2^H/^1^H array on the ^2^H phantom.

Figure 5 compares the simulated and experimental B_1_^+^ distributions of both the ^1^H transceiver array and ^2^H transmit birdcage on the ^2^H phantom, highlighting the uniform excitation profile achieved by the ^2^H transmit birdcage. The ^1^H transceiver array exhibited the characteristic CP mode at 7T, with the highest B_1_^+^ in the center and a drop‐off toward the periphery. With 1watt input power, the simulated ^2^H B_1_^+^ was 2.48 μT, while the experimental measurement was 1.46 μT in the center of the deuterated water phantom. In comparison, the ^1^H B_1_^+^ values were 0.504 μT in simulations and 0.332 μT in experimental results at the same location.

**FIGURE 6:**
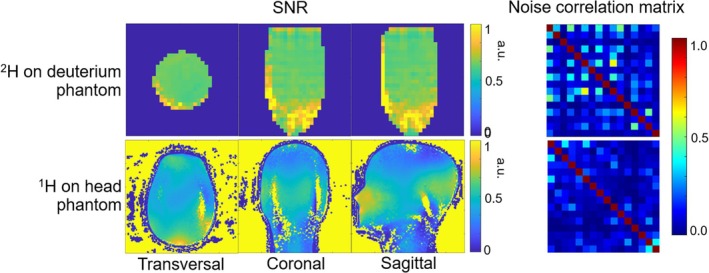
^2^H SNR of the 7T ^2^H/^1^H array on the ^2^H phantom and the ^1^H SNR on the head and shoulder phantom, along with the corresponding noise correlation matrices.

Figure 6 illustrates the SNR of the ^2^H 16‐channel receive array measured in the ^2^H phantom and the SNR of the ^1^H 16‐channel transceiver array measured from the head and shoulder phantom, along with the corresponding noise correlation matrices. The ^2^H SNR was highly homogeneous across the ROIROI, while the ^1^H SNR is highest in peripheral area. For the ^2^H receive array, the maximum and mean off‐diagonal noise correlation values were 0.61 and 0.1254, respectively, while these two numbers are 0.36 and 0.085 for the ^1^H array.

**FIGURE 7:**
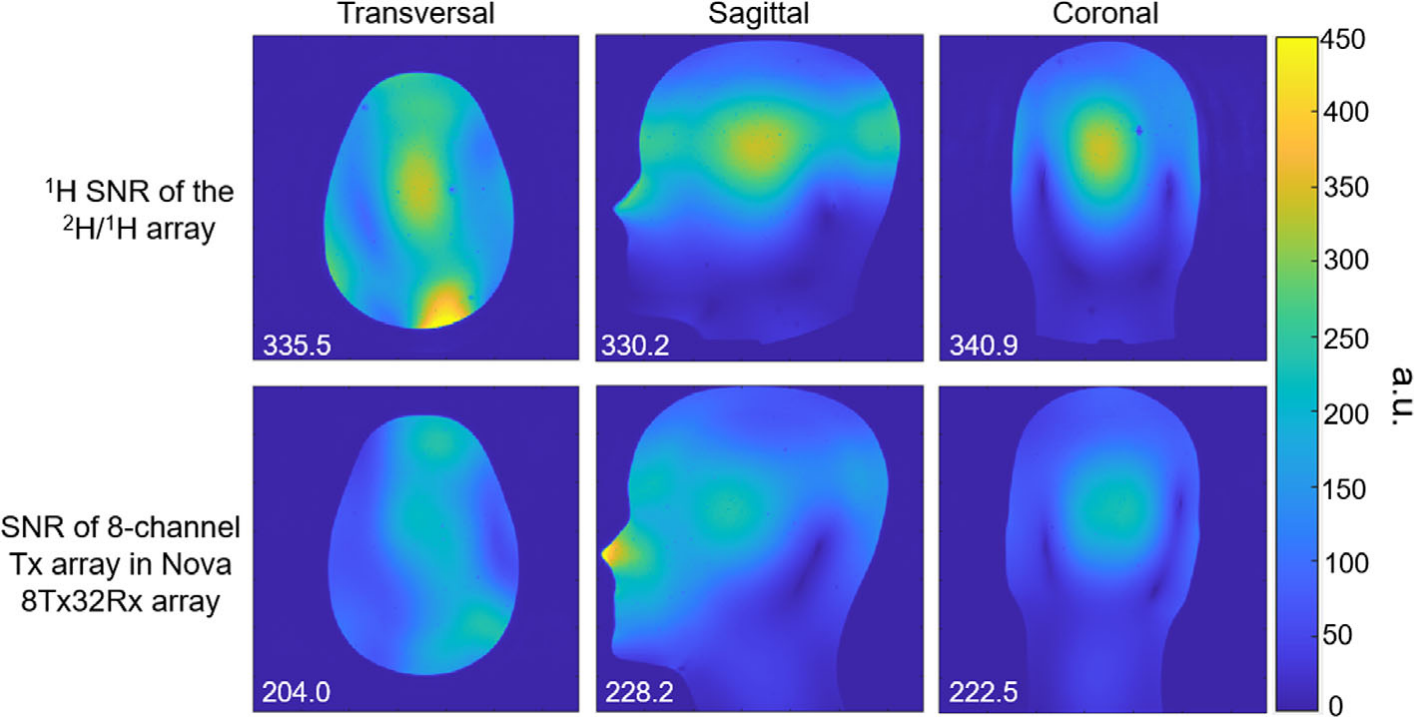
SNR of the 1H 16‐channel transceiver array and the SNR of the 8‐channel transmit array in the Nova 8Tx32Rx array (unit: a.u.).

Figure 7 shows SNR of the ^1^H transceiver array and the Nova 8‐channel transmit array, respectively, wherein the value of the central SNR is marked on the bottom left of each subfigure, In the central area, the ^2^H/^1^H array achieved 50% higher SNR than the Nova 8‐channel transmit array.

**FIGURE 8:**
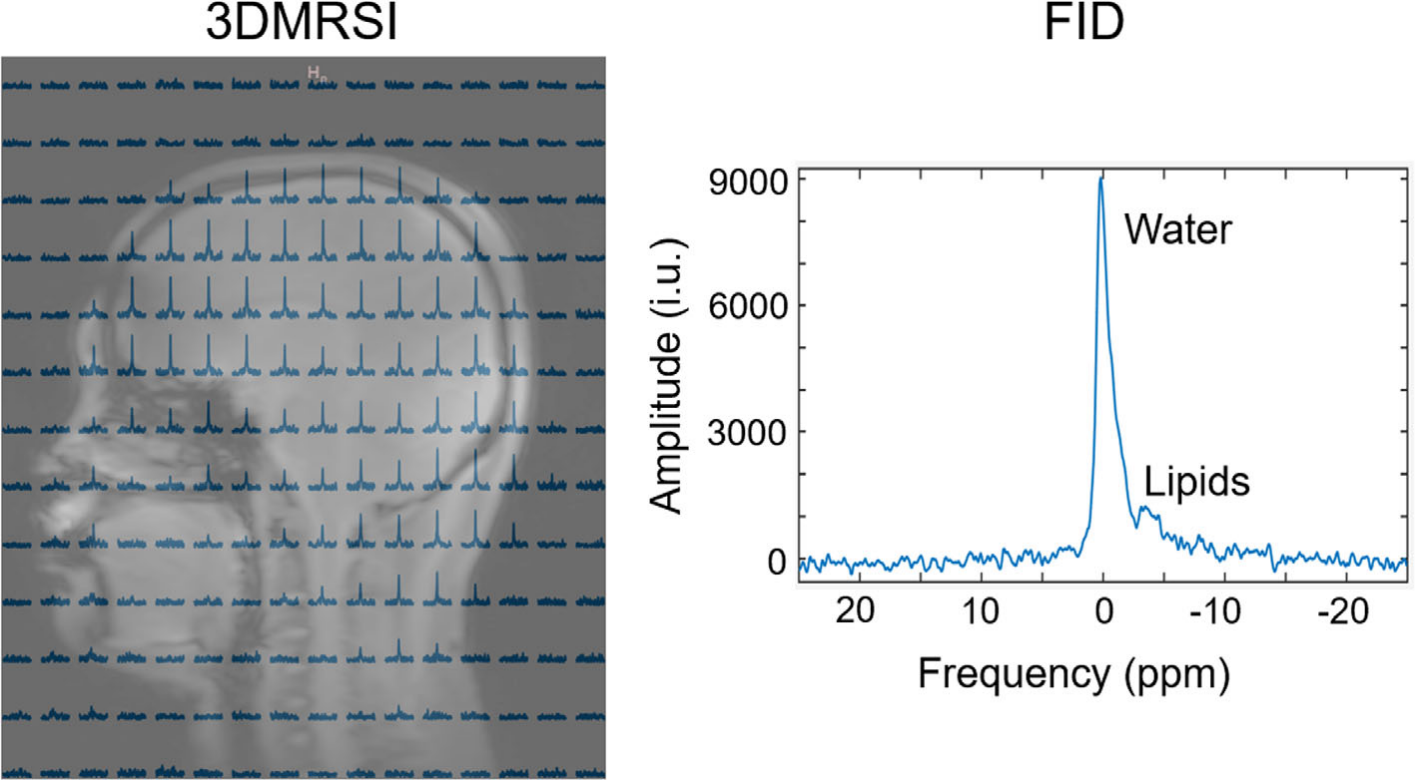
In vivo 3DMRSI spectra in the sagittal plane, showing that the ^2^H receive array has signal coverage of the entire brain; the peaks of water and lipids can be seen from a non‐localized FID acquisition.

Figure 8 presents in vivo ^2^H spectra acquired from naturally abundant deuterated water using the ^2^H 16‐channel receive array, showing ^2^H signal throughout the entire brain, including inferior brain regions. This demonstrates that the coil has signal coverage beyond the superior brain. In addition, non‐localized ^2^H FID acquisition exhibits the dominant water peak together with additional resonances attributable to lipids.

**FIGURE 9:**
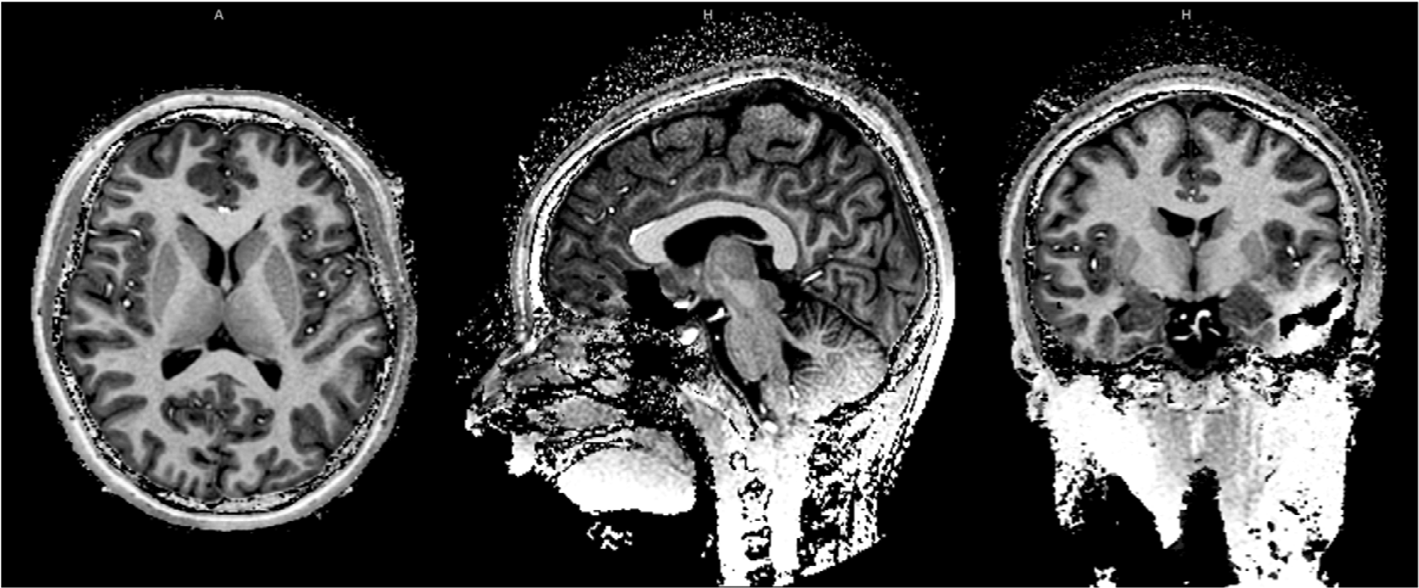
In vivo MP2RAGE images (1 mm isotropic resolution, 188 sagittal slices, SENSE = 1.6 (HF) × 2.5 (AP), FA = 5°, TI 513/3013 ms, TR = 6.2 ms, TE = 2.2 ms, BW = 162 Hz/pixel, and TA = 6 min 2 s).

Finally, Figure 9 presents MP2RAGE images with strong contrast in the transverse plane, though some signal loss was observed near the dome of the head and the cerebellum. This is likely due to the ^1^H 16‐channel transceiver array being offset by 2 cm toward the feet, combined with the potential shielding effect of the end‐ring structure of the ^2^H transmit birdcage.

## 4 Discussion

In this study, we introduced a novel 7T ^2^H/^1^H coil configuration (^2^H 1‐channel transmit/16‐channel receive array +^1^H 16‐channel transceiver array) specifically for head DMI studies. While high‐quality images from a 7T 32‐channel sodium array have been previously reported [19], the exact design and configuration of the array were not disclosed. To the best of our knowledge, this work represents the first reported 7T ^2^H 16‐channelreceive array. The decision to implement 16 ^2^H coil elements was driven by the limitation of our scanner's multinuclear receive channels to 16. However, the design presented here is adaptable to any larger number of receive coil elements supported by the system hardware.

*Q*_unload_/*Q*_load_ is reported for two configurations of the ^2^H transmit birdcage: with the ^1^H transceiver array and the ^1^H LCC traps installed, and without the ^1^H array and without traps. The configuration with the ^1^H array but no ^1^H traps could not be measured because the ^1^H elements were not tunable or matchable without the traps and removing the ^1^H traps would require extensive unsoldering/resoldering that may risk changing the tuning of the ^2^H transmit birdcage. Future designs incorporating modular or removable trap structures may facilitate full characterization of all intermediate *Q*‐factor states on workbench.

Compared to the noise correlation matrix (fig 1 in [18]) of a previously reported 15‐channel sodium array which used conventional geometrically‐decoupled coil elements in [18], the noise correlation matrix of our ^2^H/^1^H array showed significantly smaller off‐diagonal values, indicating better isolation among the coil elements. This is even in the case that the ^2^H frequency is lower than the ^23^Na frequency at 7T and no geometrical decoupling strategy was implemented in the ^2^H receive array. The improved decoupling in our ^2^H 16‐channel receive array is a result of using high‐impedance coil as receive coil elements. This aligns with the findings in [29], in which their 3T ^13^C 8‐channel high‐impedance receive array also demonstrated excellent decoupling performance.

The ^2^H transmit birdcage coil was originally designed as a bandpass configuration, with two 300 pF capacitors on each rung and two 300 and one 390 pF capacitors on each end‐ring segment, thus to ensure that each segment of the transmit birdcage was shorter than ^1^H *λ*/4. After observing that the tuning and matching of the ^1^H coil element were affected by the ^2^H transmit birdcage, the two 300 pF capacitors on the end‐ring segment were replaced by two ^1^H LCC traps (whose capacitance remains to be 300 pF at ^2^H frequency), and then another two ^1^H LCC traps were used to replace the 300 pF capacitors on each rung, at which point the interference became negligible on workbench test. Thus, the final number of traps—two on each end‐ring segment and two on each rung—was not chosen a priori but was determined empirically. Notably, a larger number of ^1^H traps were required than initially expected (i.e., the tuning/matching of the ^1^H coil element is not perturbed). This is likely due to the original 300 pF tuning capacitors on the ^2^H transmit birdcage rungs and end‐rings, which behave almost as DC blocks at the ^1^H frequency, effectively lengthening each segment to near or above a quarter wavelength at ^1^H and thus increasing coupling to the ^1^H transceiver array. Multiple traps were therefore necessary to achieve sufficient suppression of ^1^H‐induced currents. Another factor that likely contributed to the relatively large number of ^1^H LCC traps required in this design is the co‐planar placement of the ^1^H transceiver array and the ^2^H transmit birdcage. In the present implementation, both coils are mounted on the same surface, resulting in strong electromagnetic coupling between the two coils at the ^1^H frequency. If the ^2^H transmit birdcage and the ^1^H transceiver array were separated into distinct radial layers, the mutual coupling between the two coils would be reduced, and fewer ^1^H LCC traps might be sufficient to achieve comparable isolation. However, introducing additional layers would increase the overall coil diameter and prototyping complexity. Therefore, the present single‐surface configuration represents a practical trade‐off between compact geometry and electromagnetic isolation.

At high fields, SAR asymmetry may emerge from the complex interaction between array geometry, coil size, and the spatial interference patterns of the transmit fields. As reported in previous work, 22 cm‐diameter and 26 cm‐diameter 8‐channel transmit arrays at 9.4T can exhibit different SAR distributions when loaded with the same human model, the 26 cm‐diameter 8‐channel transmit arrays has higher SAR in the left orbital region [30]. In this work, the 27 cm‐diameter 16‐channel ^1^H transceiver array shows an even more pronounced localized SAR increase in the left orbital region, as can be seen in Figure 3, which may be attributed to the interference between the two rows of transmit coil elements. This observation highlights the importance of full‐wave electromagnetic simulations for evaluating transmit efficiency and SAR in UHF MRI [31].

The experimental B_1_^+^ distribution obtained from the ^1^H transceiver array was in agreement with the simulated B_1_^+^ distribution in Figure 4, while the ^2^H receive array were excluded in simulation. This consistency between simulation and experimental results further confirmed that the ^2^H receive array did not adversely affect the ^1^H B_1_^+^ field distribution, ensuring the validation of the simulated SAR results in Figure 3.

In a noise‐free simulation environment, under identical input power conditions, the ^2^H transmit birdcage achieved an approximately 10‐fold increase in B_1_^+^ in the center compared to the ^1^H 16‐channel transceiver array, as shown in the B_1_^+^ map in the Duke model in Figure 3. In the ^2^H phantom simulations in Figure 5, the ^2^H B1^+^ was 2.48 μT, while the ^1^H B1^+^ was 0.504 μT, corresponding to a 4.9‐fold ratio. The cylindrical deuterated‐water phantom used in the experiments has a diameter of only 120 mm and presents a light load. In contrast, the Duke head model has a substantially larger diameter and includes heterogeneous tissue structures. At the ^1^H frequency, the larger electrical size of the head results in increased attenuation of the transmit field with depth, leading to a more pronounced reduction of ^1^H B_1_^+^ at the center.

In the ^2^H phantom experimental result in Figure 5, the observed B_1_^+^ ratio was 4.4‐fold, slightly lower than the phantom‐based simulations (4.9‐fold). It is worth to note that there are no ^1^H LCC traps included in simulation. Given the wavelength difference between the two nuclei, power loss along the multinuclear transmit path from the multinuclear power amplifier is lower than that of the proton transmit path, 1.1 versus 2.7 dB in our system. Therefore, the power loss attributed to the ^1^H LCC traps can be estimated as

(1) $$100\%-{\left(4.4/4.9\right)}^{2}\times 10^{-\left(2.7-1.1\right)/10}=44.2\%$$

By comparing the simulated and experimental B_1_^+^ distributions in Figure 4, it is evident that achieving the same B_1_^+^ in the ^1^H transceiver array requires approximately 2.3 times of power in the experiment. However, while the experimental power measurement was taken at the output of the ^1^H power amplifiers, the simulated power was measured at the coil input. Considering that approximately half of the transmit power is lost along the transmit path from the power amplifiers to the coil input (2.7 dB), the simulated and experimental ^1^H B_1_^+^ efficiencies are in good agreement. It is important to note that the ^2^H receive array was included in the phantom experiment but was not accounted for in the full‐wave simulation, further confirming that the ^2^H receive array had minimal influence on the performance of the ^1^H transceiver array.

Deuterium SNR of the 7T ^2^H/^1^H array on the phantom shows sufficient signal coverage and receive uniformity of the array in the ROI and has been demonstrated by the ^2^H 3D MRSI spectra of the natural abundance of deuterium in the brain shown in Figure 7.

To mitigate the interference with the input 0° and 90° ports of the ^2^H birdcage, the ^1^H array was deliberately positioned 20 mm offset toward the feet of the phantom. This configuration resulted in a shift of the highest ^1^H B_1_^+^ from the corpus callosum to the thalamus and led to a slight reduction in coverage near the dome region. This can be resolved with the RF shimming capability in the parallel transmit system.

Eight 1:2 power splitters were used to interface the 16‐channel ^1^H transceiver array with the 8‐channel transmit system. The fixed 45° geometric displacement between each paired element preserves the intended CP^+^ phase progression, consistent with previous designs that adapted multichannel transceiver arrays to systems with fewer available transmit channels [32, 33, 34]. The coil can be directly adapted to a full 16‐channel pTx platform.

## 5 Conclusion

In conclusion, we have presented a novel 7T ^2^H/^1^H coil configuration consisting of a ^2^H 1‐channel transmit/16‐channel receive array, and a ^1^H 16‐channel transceiver array for deuterium magnetic resonance imaging (DMI) studies. Our design offers a significant advancement over previous multinuclear coil configurations, particularly in terms of signal quality and spatial coverage. Despite the challenges posed by the ^2^H transmit birdcage's influence on the ^1^H transceiver array, we successfully mitigated this issue through the use of multiple LCC traps, maintaining optimal performance across both nuclei. Furthermore, the coil's ability to deliver uniform B_1_^+^ fields at the ^2^H frequency and its adaptability to different receive channel configurations make it a versatile tool for high‐field MRI applications. Overall, the ^2^H/^1^H coil configuration represents a powerful new tool for multinuclear imaging, offering a promising avenue for further research and clinical applications in DMI at 7T.

## Funding

This work was supported by the Cancer Prevention and Research Institute of Texas (CPRIT) RR180056.

## Data Availability

The data that support the findings of this study are available from the corresponding author upon reasonable request.
